# Color crowding considered as adaptive spatial integration

**DOI:** 10.1167/jov.24.13.9

**Published:** 2024-12-10

**Authors:** Guido Marco Cicchini, Giovanni D'Errico, David Charles Burr

**Affiliations:** 1 Institute of Neuroscience, CNR, Pisa, Italy; 2 Department of Neurosciences, Psychology, Drug Research and Child Health, University of Florence, Firenze, Italy; 3 School of Psychology, University of Sydney, Camperdown, NSW, Australia

**Keywords:** color vision, crowding, efficient perception

## Abstract

Crowding is the inability to recognize an object in clutter, classically considered a fundamental low-level bottleneck to object recognition. Recently, however, it has been suggested that crowding, like predictive phenomena such as serial dependence, may result from optimizing strategies that exploit redundancies in natural scenes. This notion leads to several testable predictions, such as crowding being greater for nonsalient targets and, counterintuitively, that flanker interference should be associated with higher precision in judgements, leading to a lower overall error rate. Here we measured color discrimination for targets flanked by stimuli of variable color. The results verified both predictions, showing that although crowding can affect object recognition, it may be better understood not as a processing bottleneck, but rather as a consequence of mechanisms evolved to efficiently exploit the spatial redundancies of the natural world. Analyses of reaction times of judgments shows that the integration occurs at sensory rather than decisional levels.

## Introduction

Much of our visual experience is served by peripheral vision. Although we have the strong impression that the visual world is uniformly sharp, resolution and image quality decrease rapidly as we move into the periphery. Part of the falloff in resolution is due to decreasing cone density with eccentricity, but this phenomenon is insufficient to account for the rapid decline of peripheral image quality. Much of the decline can be ascribed to cortical processing, whose mechanisms remain poorly understood.

One highly studied phenomenon that limits peripheral vision is “crowding,” the inability to recognize and identify objects presented in clutter, despite their being clearly visible and recognizable in isolation (Bouma, 1970, Bouma, 1973; Herzog, Sayim, Chicherov, & Manassi, 2015; Levi, 2008; Pelli & Tillman, 2008; Strasburger, Rentschler, & Juttner, 2011; Whitney & Levi, 2011). Crowding is primarily (but not exclusively) a phenomenon of peripheral vision, well-described by Bouma's law, which states that the critical flanker–target distance to cause crowding increases with eccentricity (Bouma, 1970, Bouma, 1973; Danilova & Bondarko, 2007; Westheimer & Hauske, 1975). Crowding leads to high confidence errors (Baldassi, Megna, & Burr, 2006) and occurs between many visual features, such as orientation (Andriessen & Bouma, 1976; Westheimer, Shimamura, & McKee, 1976), spatial frequency (Chung, Levi, & Legge, 2001), color (Poder & Wagemans, 2007; Tripathy & Cavanagh, 2002) and motion (Banton & Levi, 1993; Bex, Dakin, & Simmers, 2003). It can occur between different objects (Farzin, Rivera, & Whitney, 2009; Louie, Bressler, & Whitney, 2007) and also between parts of the same object (Martelli, Majaj, & Pelli, 2005). Different features (such as color and motion) can cause crowding independently (Greenwood & Parsons, 2020), suggesting that crowding emerges from intrinsic cortical mechanisms, regardless of site of action.

While there has been much work on crowding over the past decades, there is little consensus about the underlying mechanisms. Early work has described crowding as an intrinsic “bottleneck” that cannot be overcome, either because peripheral features are pooled over space (Balas, Nakano, & Rosenholtz, 2009; Flom, Heath, & Takahashi, 1963; Freeman & Simoncelli, 2011; Greenwood, Bex, & Dakin, 2009, Greenwood, Bex, & Dakin, 2010; Parkes, Lund, Angelucci, Solomon, & Morgan, 2001; Pelli, Palomares, & Majaj, 2004; van den Berg, Roerdink, & Cornelissen, 2010; Wilkinson, Wilson, & Ellemberg, 1997; Zahabi & Arguin, 2014) or because there are access limitations that make it impossible to disentangle the target from nearby flankers (Ester, Klee, & Awh, 2014; Ester, Zilber, & Serences, 2015; Huckauf & Heller, 2002; Krumhansl & Thomas, 1977; Strasburger, Harvey, & Rentschler, 1991; Zhang, Zhang, Liu, & Yu, 2012). Recent evidence, however, is revealing a more articulated picture where crowding strength is not fixed, but is regulated in a principled manner. One of the rules that characterizes crowding is that it occurs only for similar items (same color, spatial frequency, etc), with little interaction otherwise (Andriessen & Bouma, 1976; Chung et al., 2001; Kooi, Toet, Tripathy, & Levi, 1994; Nazir, 1992; Poder, 2007; Scolari, Kohnen, Barton, & Awh, 2007). An important series of experiments by Herzog et al. has further shown that crowding can be dramatically reduced, even eliminated, when extra stimuli are added in a way to group with the flankers (Chicherov & Herzog, 2015; Herzog et al., 2015; Malania, Herzog, & Westheimer, 2007; Manassi, Sayim, & Herzog, 2012; Saarela, Sayim, Westheimer, & Herzog, 2009). They explain crowding as a grouping process, where flankers compulsorily combine with the target to create a perceptual group (Choung, Bornet, Doerig, & Herzog, 2021; Malania et al., 2007).

Crowding is generally considered to be highly detrimental to perception, particularly for many important perceptual tasks such as reading. However, much research has shown that many supposed visual deficits, such as visual illusions, are often well- explained by processes that normally lead to better perception (Gregory, 1966). Recent evidence has underlined that pooling peripheral information leads to better perceptual invariance in texture perception (Ziemba & Simoncelli, 2021), a phenomenon that itself may be flexible (Herrera-Esposito, Coen-Cagli, & Gomez-Sena, 2021). More recently, our group has suggested that crowding may be the byproduct of a system that integrates information flexibly, often leading to improved performance (Cicchini, D'Errico, & Burr, 2022).

One crucial feature to tease apart these views is that there should be circumstances when the presence of flankers can improve performance. One may expect the best chance of demonstrating flanker-induced improved performance would be when flankers have similar features to the targets. We have documented this phenomenon previously for the case of an orientation task. Here we extend the finding to color crowding. We, therefore, designed an experiment where observers had to judge the color of a peripheral patch crowded by flankers, with respect to a reference, using a similar (but not identical) paradigm to that used by Greenwood and Parsons (2020).

Because one of the hallmarks of the optimal integration theory is that integration should be weighted flexibly by the relative reliability of color information afforded by the target and the flankers, we ran the experiment with both high and low color purity of the target, predicting that the effect of flankers should be stronger for targets of low purity. To anticipate the results, we show that although the flankers strongly bias perceived hue, they can, under some conditions, also improve precision, more so for patches of low hue purity, supporting the thesis that there are specific cases where efficient integration occurs. We also reconcile our results with those of Greenwood and Parsons (2020) by a reanalysis of our data to fit their paradigm more closely.

## Methods

### Participants

Seventeen participants were recruited for the experiments, 11 female and 6 male. Two were authors; all the others were naïve to the purposes of the experiment. All participants had normal color vision (assessed by Ishihara color blindness test) and normal or corrected-to-normal vision. The observers were aged from 21 to 46 years at the time of measurement. Experimental procedures are in line with the declaration of Helsinki and approved by the local ethics committee (Commissione per l'Etica della Ricerca, University of Florence, July 7, 2020, n. 111). Written informed consent was obtained from each participant, which included consent to process, preserve, and publish the data in an anonymous form.

### Stimuli

Stimuli were programmed in MATLAB (R2016b; MathWorks) using the Psychophysics Toolbox extensions (Brainard, 1997; Kleiner, Brainard, & Pelli, 2007; Pelli, 1997). Targets and flankers were cowhide circular patches of 1.9° diameter. The target was presented 17.6° above fixation with two radial flankers. We added random jitter (±1.6° vertical axis, ±1° horizontal) to the target position to prevent color adaptation. The center-to-center distance between the target and flankers was 2.1°, corresponding on average with 0.12 times the eccentricity (within standard interference zones (Bouma, 1970; Kurzawski et al., 2023; Pelli & Tillman, 2008).

The luminance pattern was a cowhide pattern generated from random matrix, low-pass filtering it with a cutoff of 2.5 cpd, and rounding it to two values (light and dark). Luminance contrast between light and dark regions of the patches was 0.30. The slight variation of luminance creates variability along the luminance dimension to discourage observers from using luminance information.

**Figure 1.**
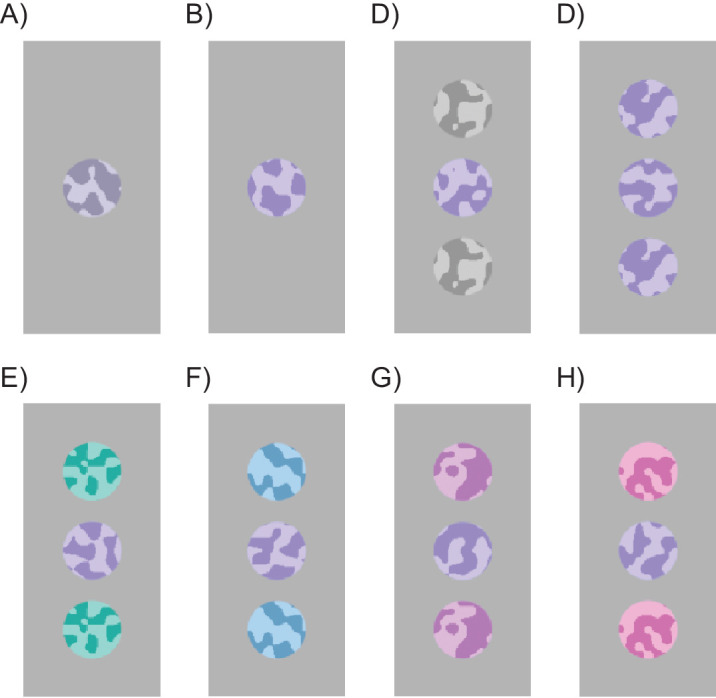
Examples of stimuli. (**A**) Low-purity (0.12) unflanked target. (**B**) High-purity unflanked target. (**C**) High-purity target with zero-purity gray flankers. (**D**) High-purity target with flankers with identical hue (Δ = 0°). (**E**) High-purity target with flanker hue greener than target, (Δ = −72°). (**F**) High-purity target with moderately greener flankers (Δ = −36°). (**G**) High-purity target with moderately pinker flankers (Δ = +36°). (**H**) High-purity target with pinker flankers (Δ = +72°)

**Figure 2.**
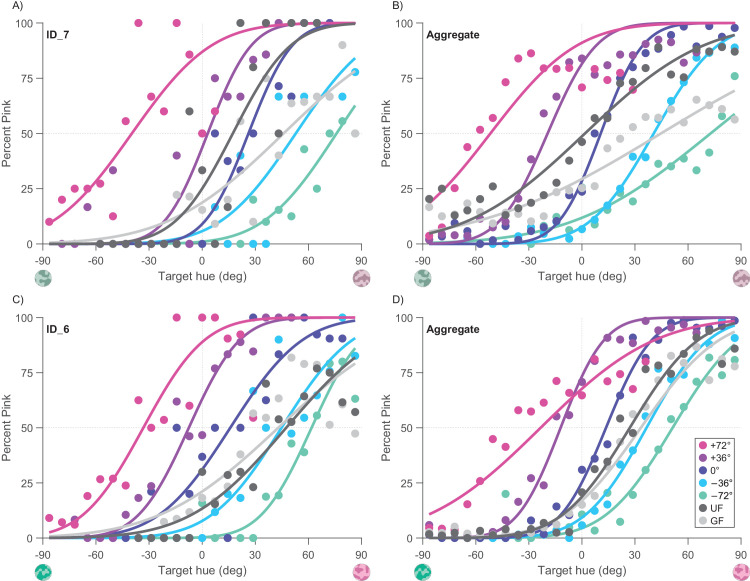
Illustrative psychometric functions. Proportion of targets judged pink as a function of target color (where 0 is the reference color). Top row (**A**, **B**) shows the results with the low-purity targets, and bottom row (**C**, **D**) shows results with high-purity targets. Color codes flanker conditions: blue and green for flankers slightly greener (Δ = −36°) or much greener (Δ = −72°) than to the corresponding target color, respectively. Mauve and pink for flankers slightly pinker (Δ = +36°) or much pinker (Δ = +72°) than the corresponding target color. Navy curves refer to a flanker with the same Derrington–Krauskopf–Lennie hue of the target (Δ = 0°). Unflanked target data are depicted in dark gray and gray flanker data (zero purity) in light gray. Please note colors in the graph do not correspond to actual flanker colors, which always varied with the target. Data and psychometric function from example observers (**A**, **C**) and aggregate observer (**B**, **D**).

Hues were defined as angles in the Derrington–Krauskopf–Lennie (DKL) color space (Derrington, Krauskopf, & Lennie, 1984; Solomon & Lennie, 2007; Witzel & Gegenfurtner, 2013). Target hues to be judged were selected from 25 equally spaced hues between 176° (green/turquoise) and 349° (pink/red). The two flankers always had the same hue, which covaried along with the target. Specifically, the differences (Δ) between their hues and the target were: Δ = 0°, ±36°, or ±72° (see Figure 1 for examples). Target hues were presented at random, and observers reported for each trial whether the target stimulus seemed to be “pinker” or “greener” than a previously learned standard, a periwinkle hue, 263° in DKL space. Plotting the percentage of “pinker” trials against target hue yielded psychometric curves like those of Figure 2.

To modulate the relative reliability of the color of target and flankers, we manipulated in separate sessions target color purity (i.e., distance from center in DKL color space). Conditions with colored flankers had an 0.34 color purity. Targets either had the same purity or 0.12 (2.8 times less). In a baseline condition, we used gray flankers with zero purity.

### Procedure

Stimuli were displayed on a linearized 27” LCD monitor (resolution 1,920 × 1,080 pixels, refresh rate 60 Hz, mean luminance 100 cd/m^2^, ΔE < 3). Observers were positioned 57 cm from the monitor, in a quiet room with dim lighting, and maintained fixation on a black dot (0.2° diameter) against a mean-gray screen.

Observers were first familiarized with the periwinkle color standard stimulus (263° in DKL space), together with examples of the target hues, with an offline session of 30 trials. They were shown the standard again, once before each session as a reminder.

Each session was self-initiated by button press, causing the stimulus to appear for 500 ms (after a random delay), followed by a blank screen that remained until response. Observers judged the target as “greener” or “pinker” than the periwinkle standard by keyboard press. They were told that all the colors spanning green to blue should be reported as “greener than the reference,” whereas violet, purple, and pink should be reported as “pinker than the reference.” Once the observer had responded, the next stimulus was displayed. No feedback was given.

In total 17 observers participated in the experiments. Nine participated in the low target purity experiment, completing a minimum of 720 trials in blocks of 60 trials (with a total of 8,076 trials and typically more than 120 trials per psychometric curve). Five participants from the first experiment together with another eight naïve observers participated in the high target purity experiment. They completed a minimum 660 trials in blocks of 60 trials each amounting to a total of 12,590 trials and more than 138 trials per psychometric curve. In both the experiments, gray flanker trials were intermingled in the same blocks with the five colored flanker conditions, while the “no flankers” trials were always in independent blocks, measured either before, after, or between subsequent “flanked” blocks. In the pilot data, we explored the possibility of intermingling also the unflanked condition with the flanked conditions. However, observers became confused by the different conditions, so we opted to run the unflanked condition separately.

### Data analysis

For each flanker condition, data were plotted as a psychometric function, plotting the percentage of “pink” responses against hue angle of the target, and fitting with a cumulative Gaussian function (see Figure 2 for examples). From the psychometric curves we derived the point of subjective equality (PSE, given by the median) and the just noticeable difference (JND; here defined as the difference in target hue angle between 50% and 84%). Up to 5% finger errors were included in the fit, allowing the error curves to saturate up to 5% and 95% (rather than 0% and 100%).

For each flanker condition, only target hues within ± 66° of the aggregate PSE (an analysis of all participants grouped together) were analyzed, for a total of 132° on the color space for each psychometric curve. Responses with reaction times more than 2.5 seconds after stimulus onset were excluded (4.8% of the analyzed trials across participants).

Although we did not ask participants to make speeded responses, they tended to do so to complete their measurements as soon as possible, allowing us to analyze reaction times, defined as the duration from stimulus onset to response. Reaction times for each flanker condition were plotted as a function of target hue, interpolating when necessary. To make the fitting more reliable we first smoothed them with a boxcar filter spanning ±14.4° of color space, fixed the lowest point of the fits to the average of the minimum response time of all flanker conditions, and limited widths of the Gaussian between 10° and 120° of color space.

We averaged the parameters calculated from all participants in each flanker condition. For illustration purposes, we display psychometric curves and response times fitting for a representative participant, and also for the aggregate participant (pooling raw data of all participants). All the other graphs refer to the group mean (mean of parameters calculated from individual participants), with standard errors as error bars.

To check whether flankers of the same hue significantly reduced response scatter, a one sample *t* test was performed for the logarithm of the ratio between JND values from the test condition (flankers with the same color of target) and the baseline condition (no flankers). A repeated measures analysis of variance was performed between the JND values of the five informative flanker conditions.

## Results

### Perceived color is a flexible average of target and flankers

Participants judged whether a color patch, presented either alone or with colored flankers, seemed to be pinker or greener than the 263° periwinkle reference patch (Figure 1). The target was either of low (Figure 1A) or high (Figure 1B) purity. Flankers were either gray (Figure 1C) or of high purity, with hue differing from target hue by one of five values (Δ = −72°, −36°, 0°, +36°, +72°) (Figures 1D–H).

Figure 2 shows the psychometric functions for the task, plotting proportion judged “pink” as a function of target color, for tests of the target alone (dark gray symbols) or with various flankers. The top row shows results for low-purity targets, the bottom high-purity targets. Curves on the left show a representative observer, at right the aggregate observer (raw data of all participants grouped together). The five flanker conditions are shown with different colors, ranging from Δ = −72° (flanker greener than target, indicated in green) to Δ = +72° (flanker pinker than target, indicated in pink). Control conditions (no flanker and gray flankers) are shown in dark and light gray, respectively.

From each psychometric curve, we extracted two key parameters, the PSE and JND. The PSE is the median of the cumulative Gaussian curve and corresponds to the point where the target looked equivalent to the reference color. Flankers clearly influenced the judgments of the target color, with pinker flankers (Δ = +36° or +72°) inducing a proportionate leftward shift of the psychometric curve, meaning that the target looked pinker than it was: an assimilative effect. Greener flankers (Δ = −36° or −72°) induced a proportionate rightward shift, again an assimilative effect.

**Figure 3.**
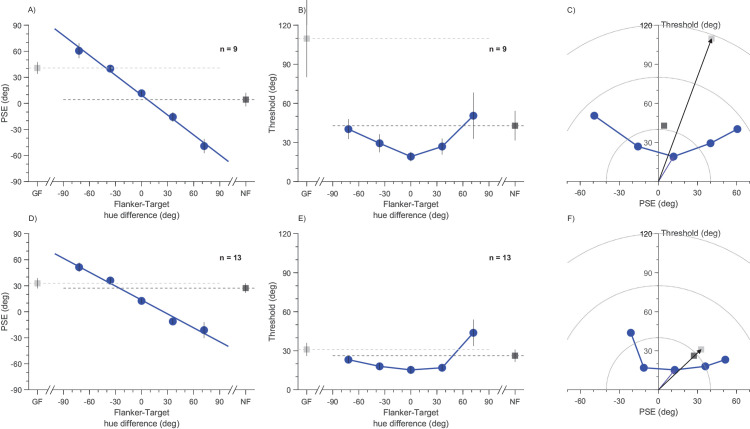
Effect of flankers on points of subjective equality (PSEs) and jut noticeable differences (JNDs). Group mean parameters for low target purity (top row) and high target purity (bottom row). (**A**) Group mean PSEs for low-purity targets as a function of flanker–target color difference (Δ) and the two baseline conditions, unflanked (NF, in dark gray) and gray flankers (GF, in light gray). The continuous line shows the linear fit for the colored flanker conditions. (**B**) Group mean JNDs as a function of flanker-target color difference. (**C**) Scatter plot of inverse precision (JNDs) against accuracy (PSE). Five conditions with colored flankers are in blue, target alone in dark gray, and gray flankers in light gray. The overall root-mean squared error (RMSE) is the distance from the origin. The two arrows emphasize the improvement in overall RMSE for the color flankers of matched hue compared with the zero-purity flankers. (**D**–**F**) as for (**A**–**C**), for high-purity targets. Error bars represent standard error of mean.

To quantify these effects, Figures 3A and 3D show the group mean PSEs (average of individual participant PSEs) for the five flanker conditions as a function of the difference in flanker and target hue (Δ). The relationship is nearly linear for both the low and high target purities, with respective slopes of −0.76, 95% confidence interval, −1.03 to −0.49, and −0.53, 95% confidence interval, −0.91 to −0.16. If the slopes were unity, it would mean that the judgments were based entirely on the flanker hue, ignoring the target. That they are less than unity (but more than zero) implies that judgments were based on a perceptual merger of the target and the flankers, with the flankers accounting for 76% of target color perception at low purity conditions and 53% at high.

### Similar flankers cause an increase in precision

A second parameter returned by the psychometric function is the JND. The JND refers to the precision associated with the two-alternative forced choice task: steep curves correspond with high precision and shallow curves with low precision. Traditionally, the JND is defined as the color difference between 50% and 84% responses, corresponding with the standard deviation of the fitted Gaussian. Inspection of Figure 2 shows that the curves with colored flankers are generally steeper than the two control conditions (gray or no flankers), implying more precise judgments.

Figures 3B and 3E plot group mean JNDs for the five flanker conditions, along with the baselines. The curves are U shaped: two separate repeated measures analyses of variance showed that the difference between flanker conditions was significant for the high target purity, F(4, 48) = 5.37, *p* = 0.001, being lowest when flanker color was matched to the target. However, it was not significant for the low-purity condition, F(4, 32) = 2.42, *p* = 0.068. Importantly, the JNDs with identical flankers were lower than for the unflanked condition, both in the low-purity condition, ratio 0.45, one-tail *t*(8) = −3.25, p= 0.006; Log10 (Bayes factor) = 1.04, and the high-purity condition, ratio 0.58, one-tail *t*(12) = −3.05, *p* = 0.005; Log10(BF_10_) = 1.06.

Figures 3C and 3F plots the two types of errors, one against each other. The response scatter is given by the standard deviation of the fitted cumulative Gaussian, which we take as JND. The bias is given by the difference between the PSE and reference. Scatter and bias are independent components of total error, with their Pythagorean sum (square root of sum of squares) corresponding with the total root-mean squared error (RMSE), a generic metric to assess overall performance. In Figures 3C and 3F, this parameter is given by the distance from the origin. It is obvious that the shortest distance, corresponding with the lowest RMSE, was for flankers equal to target (Δ = 0°): for the low-purity targets RMSE with equal flankers is 0.52 times the unflanked threshold, one-tail *t*(8)= −3.17, *p* = 0.007, Log10(BF_10_) = 1.01; for the high target, the purity RMSE was also 0.52 times the unflanked condition, one-tail *t*(12)= −3.12, p= 0.004, Log10(BF_10_) = 1.10. In the low-purity condition, all flanker conditions had a lower RMSE than the gray-flanker condition.

**Figure 4.**
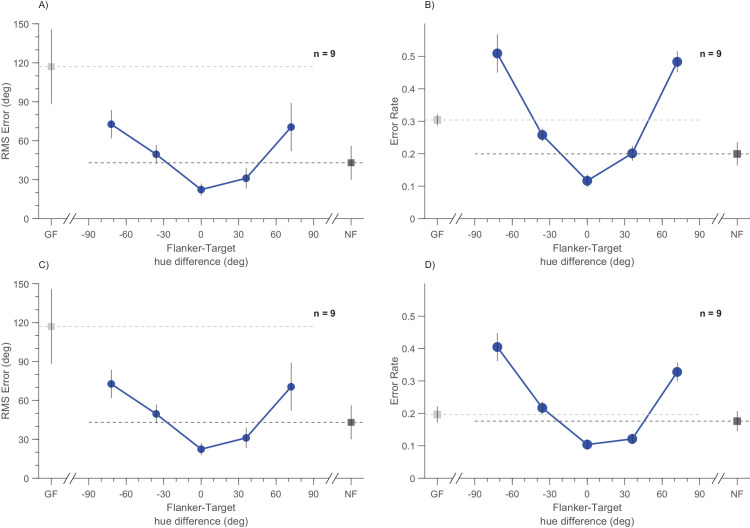
Error rate measured in two different ways. (**A**) Group means of root-mean square error (RMSE) for the low-contrast condition as a function of flanker–target color difference. Gray flankers are plotted in light gray and unflanked in dark gray. Data plot the distance from the origin of the datapoints of Figure 3. (**B**) Group means of error rate across all trials as a function of flanker-target color difference. Lower values indicate better performance. (**C** and **D**) Same as (**A** and **B**), but for the high target purity condition. All other conventions like Figure 3.

Total error (the norms of the polar plots of Figures 3C and 3F) is replotted in cartesian coordinates in Figures 4A and 4C. Again, this error is minimal for flankers identical to targets, then increases with target-flanker difference. For both low- and high-purity conditions, total error is similar or lower than the unflanked condition for the three central points, but rises above the control for the extreme flankers, 72° different from the targets.

With a few exceptions, crowding studies do not usually differentiate between bias and precision, but simply measure overall accuracy, with a measure such as “error rate.” Figures 4B and 4D show our results reported as error rate (essentially all responses in the top right and bottom left quadrants of Figure 2 are correct, the others are errors). Also by this measure, when the flankers are the same or similar, the error rates do not change dramatically and may even improve. For larger differences, the error rates soar. So by most standard tests of crowding, the flankers, unless similar to the target, severely deteriorate performance.

### Reaction times analysis

Biases in psychometric curves could be generated either at the level of signal processing, reflecting a genuine perceptual bias, or can result from response biases (such as “if unsure say ‘greener’”). One way to disentangle the two effects is to look at response times (Maldonado Moscoso, Cicchini, Arrighi, & Burr, 2020). The logic is explained in detail in the publication by Maldonado Moscoso et al. (2020), but briefly, targets at or near the point of subjective equality should seem to be very similar to the standard, so the judgments are difficult, leading to longer response times for those conditions. If the peaks in response times (reflecting difficult decisions) move with flanker condition like the PSEs, that would be evidence for action at perceptual rather than decisional levels, implying that the flankers interact directly with the target to cause it to appear like the standard. In contrast, if the flankers act at the decision level, biasing only the response, there is no reason why the peaks in reaction times should change.

**Figure 5.**
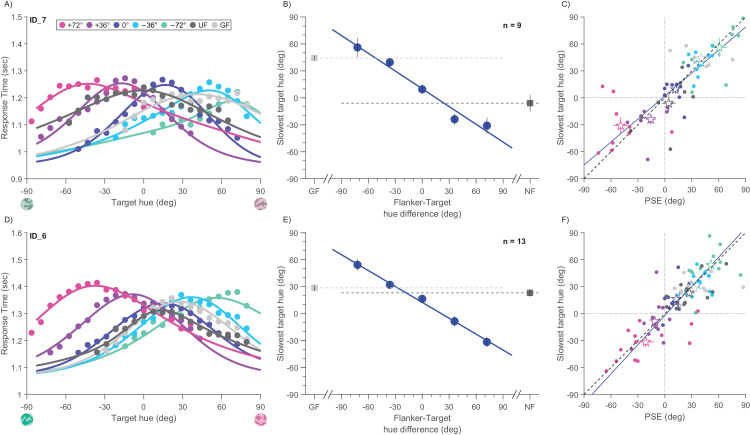
Response time analysis. Illustrative observer and group mean parameters for low target purity (top row) and high target purity (bottom row). (**A**) Gaussian fit of mean response time as a function of target hue for the five flanker–target color differences (Δ), gray flankers (light gray), and unflanked (dark gray). Peaks of response time are shifted to greener for pinker flankers and vice versa, as with to points of subjective equality (PSEs). Color code as in Figure 2. (**B**) Group mean of Gaussian fit peaks as a function of flanker-target color difference. (**c**) Scatter plot of peaks of the Gaussian fit to RT against PSE for each participant and condition. Color codes flanker–target color difference as in Figure 2. Group mean depicted with star symbol. Continuous lines indicate best linear fit between PSEs and slowest hue: R = 0.83 and *p* < 0.001 for low target purity targets. (**D**–**F**) As for (**A**–**C**), for high-purity targets. Best linear fit between PSEs and slowest hue (**F**): r = 0.84 and *p* < 0.001. Error bars represent standard error of mean.

Figures 5A and 5D plot response times as function of target hue for the observers shown in Figures 2A and 2D. The response times for all five conditions were well-fitted by Gaussian curves. Clearly, the peaks in response times were not the same in all conditions, but are displaced systematically, in the same direction as the PSEs of the psychometric functions.

Figures 5B and 5E plot the peaks of the Gaussian fits (like those of Figures 5A and 5D), averaged over all observers. The pattern of results resembles closely the results of PSEs calculated from the psychometric curves (Figure 3), with pinker flankers shifting the peaks in the green color direction, and greenish flankers toward pink. In this case, the slope of linear fit is −0.66, 95% confidence interval −0.98 to −0.34, for the low target purity and −0.59, 95% confidence interval −0.94 to −0.24, for the high.

Figures 5C and 5F plot the hue that yields slowest responses against PSEs (from Figure 3), both for individual observers and for the group mean (star symbols). Most datapoints lie close to the diagonal, indicating a tight link between the two quantities, r = 0.83, *p* < 0.0001 for low purity and r = 0.84, *p* < 0.0001 for high purity. The response times suggest that the shifts in PSE reflect genuine perceptual changes, rather than response biases.

**Figure 6.**
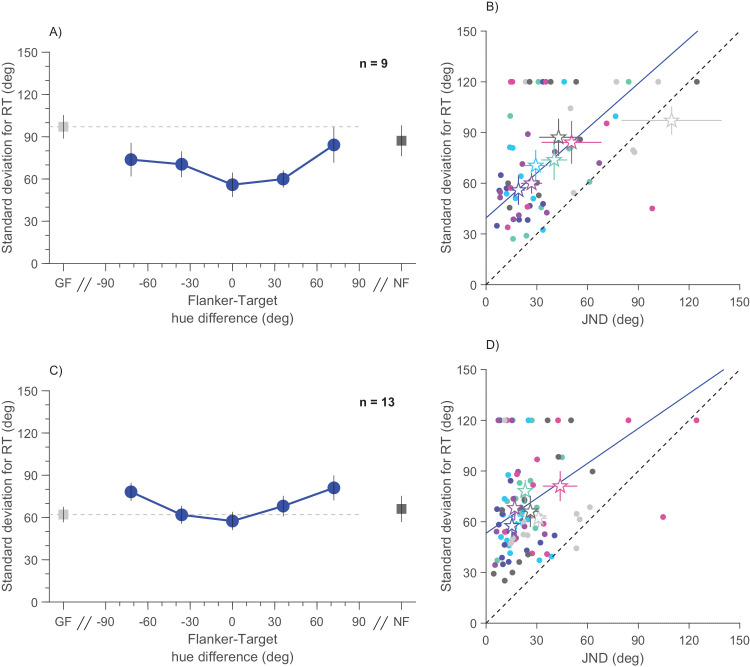
Widths of RTs. (**A**) Group mean of Gaussian fit widths as a function of flanker-target color difference. (**B**) Scatter plot of widths of the Gaussian fit to RTs against the just noticeable difference (JND) for each participant and condition. Color as in Fig. 2. Group mean depicted with star symbol. Continuous lines indicate best linear fit between width of RT fits and JNDs: r = 0.38 and *p* = 0.009 for low target purity targets. (**C**–**D**) As for (**A**–**D**), for high-purity targets. Best linear fit r = 0.26 and *p* < 0.037. Error bars represent standard error of mean.

It is apparent from inspection of Figures 5A and 5D that the widths of the Gaussian fits also vary with condition. The widths of the RT curves indicate the range of stimuli with a slow response, reflecting that stimuli within that range are confusable with the reference. Figures 6A and 6C plot the average standard deviations of the curves as a function of flanker-target difference. The curves follow the same general shape as the JNDs (Figures 3B and 3E), with the result for matched target and flankers below the unflanked condition, low purity *t*(8)=2.83 and *p* = 0.02, high purity *t*(12)= −0.7 and *p* = 0.46. Indeed, the standard deviations correlate well with the JNDs (Figures 6B and 6D), r = 0.38 and *p* = 0.009 and r = 0.26 and *p* < 0.037 for low- and high-purity targets, respectively. The standard deviations of the RTs were not identical to the JNDs (generally greater), but clearly related. This finding reinforces the claims that precision is higher with some flanker conditions than unflanked targets, even when measured by this very indirect means, and again points to a sensory interaction, rather than response bias.

### Reconciliation with Greenwood and Parsons (2020)

Perceptual improvement in the face of color crowding may seem at odds with previous literature showing crowding-induced threshold elevation (Greenwood & Parsons, 2020). Although the paradigm of that study was one of the few to distinguish between flanker bias and precision, there are major paradigm differences between the two studies. Most notably, Greenwood and Parsons induced maximal crowding with flankers of fixed hue at a color similar to that of the reference color. In contrast, our flankers were yoked to the target color, trial by trial. To check whether this can yield radically different results, we extracted from our dataset conditions where the various targets were paired with a flanker with color similar to the reference (263° ± 7°), comparable with the conditions of Greenwood and Parsons (2020).

**Figure 7.**
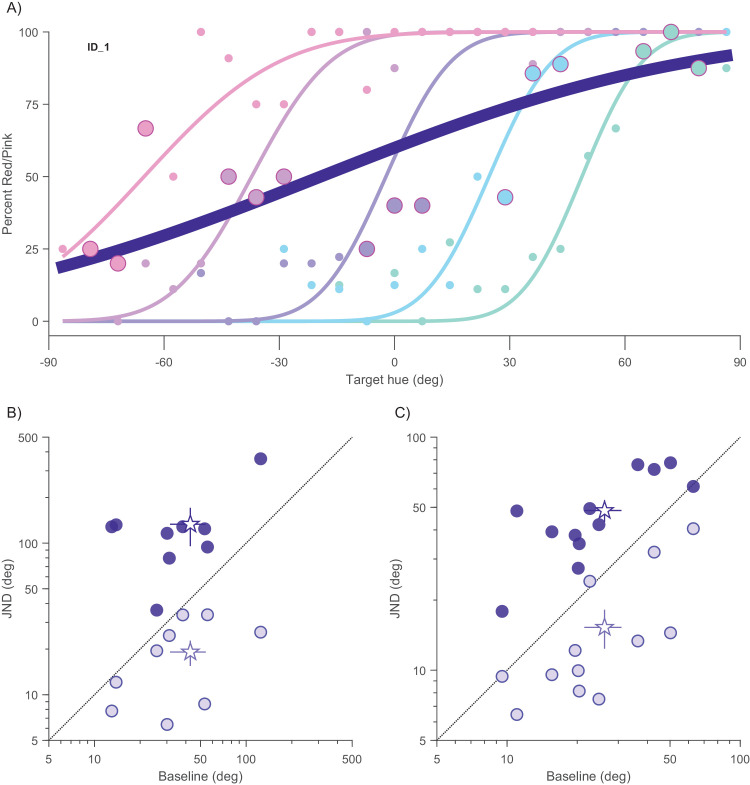
Extraction of hue fixed flanker condition. (**A**) Subdued-colored data and psychometric functions from the observer G.M.C. with the five colored flanker condition for the low-purity target. From each curve points where flankers were of the reference periwinkle are highlighted. The obtained psychometric function, much shallower than the five others is shown in dark violet. Just noticeable differences (JNDs) with flankers equal to target (Δ = 0°, hollow circles) and with fixed hue flankers (solid circles) as a function of the unflanked JNDs (baseline) for each participant with low target purity (**B**) and high target purity (**C**). Group mean depicted with star symbol. Error bars represent standard error of mean.

Figure 7A illustrates this principle. Even with curves that contain pinker flankers (+72° with respect to the target, depicted in subdued pink), there are some trials in which the absolute color of the flanker was similar to the reference color: this point would belong to a curve of maximal crowding (0° flankers in their terminology) with target hue at −72°. We repeated this operation for all five psychometric curves, isolating those points where the flankers were similar to the reference color. In this way, we were able to fit a psychometric function on the same data sample, simulating the effect of an absolutely defined flankers. In one observer, the predicted JND was above 360° (impossible), and therefore capped at 360°. Figures 7B and 7C plot JNDs collected in the two critical crowding conditions, one with flankers identical to target (Δ = 0°) in light violet, the other with “fixed flankers of reference periwinkle” in dark violet, as a function of the baseline JND (collected without flankers).

The plot not only shows how in our conditions nearly all participants lie below the diagonal, but it also displays that the same dataset, if rearranged so to pool in one psychometric curve trials that had the same flanker, one would obtain a strong threshold elevation, low target purity: near seven-fold, one-tail *t*(8)= −6.54, *p* < 0.0001, Log10(BF_10_) = 5.84; high target purity: near three-fold, one-tail *t*(12) = −9.09, *p* < 0.0001, Log10(BF_10_) = 10.4.

**Figure 8.**
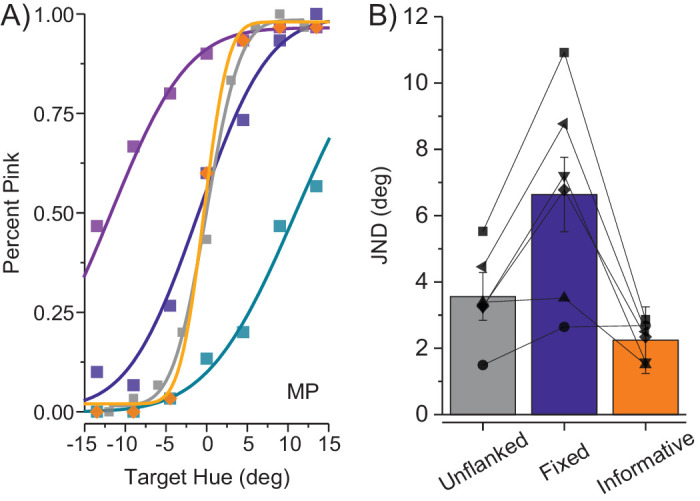
Reanalysis of data from Greenwood and Pearson (2020). (**A**) Proportion of targets judged pink as a function of target color for an illustrative participant (where 0 is the reference color for the participant). Color codes flanker conditions: gray for unflanked target, purple for target flanked by a patch slightly pinker than the reference (+15° CCW), turquoise for the opposite (−15° CW from reference), and violet for flankers placed at the reference color. In the orange, we plot the psychometric curve obtained using same-sign flankers, where target and flanker had the same sign of relative hue angle. (**B**) Just noticeable differences as a function of flanker condition: unflanked from Greenwood and Parsons’ dataset in dark gray, fixed flankers equal to reference color as in Greenwood and Parsons (2020) in violet and same-sign flankers in orange. Group mean is presented along with the standard error of the mean and individual data are plotted with black symbols. Thanks to J. Greenwood for providing data.

Some minor experimental differences exist between our paradigm and that of Greenwood and Parsons. For instance, in their work flankers could only have one of two colors within one session. So a good test would be to generalize our approach to the data collected by Greenwood and Parsons (2020) with their setup and instructions. Unfortunately, the original dataset did not lend itself to a full reconstruction of the psychometric functions, because there were only three target hues that were tested with a similar flanker hue, too few to draw a full psychometric function. However, we isolated trials where the target was accompanied by a potentially “informative flanker”: for targets greener than the reference, we selected trials where flankers were also greener than the reference; and for pinker targets we selected pinker flankers. The procedure is illustrated in Figure 8A, where the purple curve corresponds with a fixed flanker that is slightly pinker than reference (+15° CCW) and the green curve to a slightly green flanker (−15° CW). To obtain a curve where flankers are potentially informative one needs to draw data from the greenish flanker curve when targets are greenish (i.e., <0°) and from the purple curve when targets are pinkish (i.e., >0°). As Figure 8A shows, the resulting psychometric function is steeper than the fixed flanker condition (violet) plotted by Greenwood and Parsons (2020). This was done for all subjects considering as greenish flankers fixed at +15 or +30 CCW and pinkish flankers those fixed at +15 and +30 CCW and shown in Figure 8B. The condition with the informative flankers is steeper respect to the fixed flanker, one-tail *t*(5)=4.5, *p* = 0.003, Log10(BF_10_) = 1.26, and also is steeper than the unflanked condition collected by Greenwood and Parson, one-tail *t*(5)=1.97, *p* = 0.053, Log10(BF_10_) = 0.35.

## Discussion

Crowding is a pervasive visual phenomenon that limits visibility in the periphery in crucial ways. This study suggests a novel interpretation of crowding: that it is a byproduct of Bayesian-like processes that aim to make perception more efficient by minimizing perceptual error, at the cost of causing distortions of perceptual input, which can be debilitating for fundamental processes such as reading.

The idea that crowding may be related to integration, which can be beneficial to some forms of perception (such as texture discrimination) has been expressed by others (e.g., Parkes et al., 2001; Ziemba & Simoncelli, 2021). However, the idea floated here is slightly different in that it suggests that the integration should not be automatic, but flexible and efficient, following Bayesian principles. This strategy leads to two testable predictions: that crowding should be strongest for targets of low reliability and that, under certain conditions, it may result in greater precision. The perceived hue of the target was strongly biased toward that of the flankers, more so for low-purity (unreliable) than for high-purity targets. The results were well-explained by the final perception being a weighted average of target and flankers, with the weight of the flankers being 76% for low-purity targets and 53% for high-purity targets. The fact that the weight of the flankers depends on the target strength shows that it is not a simple form of compulsive pooling. Interestingly, several previous studies have found that the relative contrast of gray-scale targets and flankers stimuli determined crowding strength (Agaoglu & Chung, 2017; Chung et al., 2001; Pelli et al., 2004). In this light, our work suggests that, rather than sheer signal strength, the crucial determinant of this effect might be relative target reliability.

A major signature of efficient integration is that it should improve overall performance, measured as a decrease in RMSE, at least under some conditions. Consistent with this idea, we found that judgments made with similar flankers were accompanied by steeper curves than those of baseline conditions. This strategy led to lower estimates of the JND (higher precision), which counterbalanced the decreased accuracy (bias errors) to yield lower estimates of total root mean squared error (Figures 3C and 3F and 4A and 4C). The effect was particularly striking for low-purity targets: unflanked targets (which were resolved poorly) had shallow psychometric curves, but those with flankers had much steeper curves, with standard deviations halved. That flanker can, under some conditions, improve precision is not consistent with many theories of crowding, likening it to a bottleneck.

Most averaging models of crowding predict improvement of precision, as the process of averaging several sources reduces variance, particularly in the representation of poorly resolved targets. What distinguishes our model from classical averaging models (e.g., Parkes et al., 2001) is its flexible nature: a fixed pooling system would blend a good target with a poor flanker, just like it would do when there is to blend a poor target with a good flanker, resulting in nonoptimal fusion. The model we suggest predict flexibility as function of target and flanker salience, as observed here.

Another signature of optimal integration is that it should be stronger for stimuli that are similar in that dimension (Cicchini, Mikellidou, & Burr, 2018). In our previous study (Cicchini et al., 2022), we verified that this was the case for orientation crowding. Here, we did not measure crowding as a function of chromatic similarity explicitly, but a wealth of data suggests that color crowding is strongest when the hues are most similar with interactions between flankers and target seem to decrease beyond 90° in DKL space (Greenwood & Parsons, 2020; Kennedy & Whitaker, 2010). Interestingly the optimal model predicts that in the case of high resolution targets the region of interaction should be smaller than in the case of poor resolution targets (for instance, see Cicchini et al., 2022). It will be interesting to verify these predictions with a dedicated experiment in the future.

It is also interesting to note that while the effects are stronger for low-purity targets, the high-purity targets also show significant reduction in response scatter, even when the target and flanker hues were matched. In this condition, the target and two flankers were identical, facilitating flanker–target substitution (see Krumhansl & Thomas, 1977; Popple & Levi, 2005; Strasburger, 2005; Wolford, 1975), but this factor could not explain the increase in steepness of the psychometric functions: confusing the target with an identical flanker should not bring any advantage. In contrast, the data are consistent with integration between flankers and target. In the ideal case that observers considered the flankers identical to the target and judged the combined ensemble of three objects as a whole, one would expect a drop of JNDs by a factor 1/$\sqrt{3}$ (= 0.58), close to the factor of 0.53 estimated by the current data.

Similarly, if the flanker–target substitution were not total, but probabilistic (occurring on a fraction of trials). it would not explain all the data. It could certainly account for the shift in PSEs toward the flanker hue: slopes of PSE against flanker (0.53 and 0.76) are consistent with sampling the flankers on 53% of high purity trials and 76% of low-purity trials. However, although a multiplexing strategy is consistent with shifts in PSE, it would necessarily lead to broad psychometric curves, as response to both target and flankers—each with different PSEs—would blend into a single broad psychometric function. In our dataset, not only do curves not become broader, they are generally narrower than for the target-alone condition.

Color perception occurs over a cascade of processes in the visual system and a crucial question when dealing with averaging of color is the underlying space where the operation occurs. In our interpretation of the data, we assumed, as commonly is done, that observers blended the hues of the stimuli and thus operate independently along the two polar coordinates of the DKL space, namely, hue and purity (Robinson & Brady, 2023). However, a theoretical alternative exists: observers might average the cartesian components of the DKL space (L vs. M opponency and L + M vs. S), similar to what is assumed for two-dimensional motion signals (Nichols & Newsome, 2002; Treue, Hol, & Rauber, 2000). Interestingly averages in this space make similar predictions to the data presented here. High-purity flankers exert a stronger influence on low-purity targets than on targets with the same purity. Also, computations in this space predict (like all integration models) that similar stimuli should display the lowest noise levels. So how to disentangle between the two hypotheses? One possibility would be to run a dedicated study asking observers to reproduce both hue and purity of the target as done in other color studies (e.g., Hansen, Olkkonen, Walter, & Gegenfurtner, 2006). Vector averaging predicts a loss of perceived purity when colors of similar purity do not have the same hue; polar averaging does not.

In this study, we have manipulated color reliability by changing color purity in DKL space. Because purity is changed also, perceived saturation also changes. At present, we cannot disentangle which of the two is contributing most to the switch in the crowding strength. Interestingly different colors of same purity may yield different perceived saturation (Indow & Stevens, 1966; Schiller, Valsecchi, & Gegenfurtner, 2018). Future studies may exploit this dissociation to understand whether the effect is mostly dependent on purity or at the later perceptual stages that compute saturation.

Analysis of response times suggests that the influence occurs at a perceptual, or sensory level, and is not merely a response bias (Gallagher, Suddendorf, & Arnold, 2019; Maldonado Moscoso et al., 2020). This finding is in strong agreement with evidence that optimal integration over time (serial dependence) occurs at early sensory levels (Cicchini, Benedetto, & Burr, 2021; Cicchini, Mikellidou, & Burr, 2017; Cicchini, Mikellidou, & Burr, 2023). It also speaks against some theories of crowding, such as probabilistic substitution, which would not lead to a single peak in reaction times.

We can also account for the fact that crowding, including color crowding (Greenwood & Parsons, 2020), is usually associated with threshold increases, even when measured as precision rather than total error. With our same dataset, we were able to replicate Greenwood and Parson's (2020) results, who locked the flanker to a specific hue while varying that of the target. If the observer integrates the flanker with the target, any manipulation of the target will be attenuated by the presence of the fixed flankers. Thus, the flankers should dilute the hue variations of the target, leading to an elevation of threshold. The only condition that should lead to an improvement of performance is when the relative difference between target and flanker is kept constant across the psychometric curve, or is at least always in the same direction. Indeed, when we reanalyzed Greenwood and Parson's data, so that flankers always had the same sign as the target (relative to the standard), their data also showed that flankers can improve performance (Figure 8).

This study reinforces our previous suggestion that crowding can be associated with efficient behavior (Cicchini et al., 2022), and broadens the conclusion that it may emerge from mechanisms aiming to minimize error, for color as well as orientation processing. The suggestion of flexible integration over space is analogous to processes that occur over time, termed “serial dependence” (Cicchini et al., 2018; Cicchini et al., 2023; Fischer & Whitney, 2014; Manassi, Murai, & Whitney, 2023; Pascucci et al., 2023). Perception of many attributes, including orientation (Cicchini et al., 2017; Fischer & Whitney, 2014; Fritsche, Mostert, & de Lange, 2017), numerosity (Cicchini, Anobile, & Burr, 2014), facial identity (Liberman, Fischer, & Whitney, 2014; Taubert, Alais, & Burr, 2016); even beauty (Alexi et al., 2018) and perceived body weight, are biased toward previously viewed stimuli. These biases in perception have been shown to reflect an efficient perceptual strategy, exploiting temporal redundancies in natural viewing to reduce overall reproduction errors, despite the biases (Cicchini et al., 2018). Here we suggest that similar processes occur over space, following very similar rules: the integration is weighted by stimulus reliability, it decreases when flanker and target are different, and, most important, results in reduced error. There are other similarities too, such as that crowding does not occur for stimuli suppressed from visual awareness (Wallis & Bex, 2011), as serial dependence does not occur for stimuli suppressed during binocular rivalry (Fornaciai & Park, 2018; Kim, Burr, Cicchini, & Alais, 2020). All this evidence points to a general strategy of attempted noise reduction, in both time and in space, and probably at many levels of processing. In this perspective, the improvement of performance reported here is an (often unwanted) consequence of general pooling principles.

To be clear, we are not suggesting that crowding is typically advantageous to humans: on the contrary, it is a major impediment to peripheral viewing, especially for tasks requiring discrimination of detail, such as letters embedded within words. However, there is a good deal of evidence showing that the system can perceive flanked targets accurately in some conditions: for example, by adding additional flankers, causing the flankers to group into an object distinct from the target (Malania et al., 2007; Manassi et al., 2012). Crowding is not inevitable, inconsistent with a processing bottleneck. Our evidence that some aspects of perception, such as precision of response (a common measure of threshold), can actually improve under certain conditions, suggests an alternative framework for understanding crowding: that it is the unwelcome byproduct of an otherwise efficient mechanism exploiting spatial redundancies of the visual scene. The redundancies may well facilitate some tasks, such as texture perception, at the expense of others, such as reading. It may well be that, during most of evolution, texture perception was more fundamental than tasks that are essential in modern times, such as reading.

In conclusion, although we agree that visual crowding remains a fundamental limitation to object recognition, we believe that it is best understood not as an insurmountable bottleneck of the system, but rather as the consequence of mechanisms aiming to exploit the spatial redundancies of the natural world (as they exploit temporal redundancies), which at times may become overzealous and overpool features that should remain segregated perceptually.
